# Effect of Brewing Duration on the Antioxidant and Hepatoprotective Abilities of Tea Phenolic and Alkaloid Compounds in a *t*-BHP Oxidative Stress-Induced Rat Hepatocyte Model

**DOI:** 10.3390/molecules200814985

**Published:** 2015-08-17

**Authors:** Laura Braud, Ludovic Peyre, Georges de Sousa, Martine Armand, Roger Rahmani, Jean-Michel Maixent

**Affiliations:** 1 Laboratoire PROTEE, EB2M, Université de Toulon, CS 60 584, 83 041 Toulon Cedex, Campus La Garde, France; 2 UMR 1331 TOXALIM (Research Centre in Food Toxicology), Institut National de la Recherche Agronomique (INRA), Laboratory of Xenobiotic’s Cellular and Molecular Toxicology, 400 Route des Chappes, 06903 Sophia-Antipolis, France; 3 Aix-Marseille Université, CNRS, CRMBM UMR 7339, F-13385 Marseille, France

**Keywords:** *Camellia sinensis*, tea, polyphenols, bioaccessibility, antioxidant, EGCG, hepatocytes, ROS, mitochondrial membrane integrity

## Abstract

Tea is an interesting source of antioxidants capable of counteracting the oxidative stress implicated in liver diseases. We investigated the impact of antioxidant molecules provided by a mixture of teas’ leaves (green, oolong, pu-erh) after different infusion durations in the prevention of oxidative stress in isolated rat hepatocytes, by comparison with pure epigallocatechin-3-gallate (EGCG), the main representative of tea catechins. Dried aqueous tea extracts (ATE) obtained after 5, 15 and 30 min infusion time were characterized for total polyphenols (gallic acid equivalent), catechins, gallic acid and caffeine (HPLC-DAD/ESI-MS) contents, and for scavenging ability against 2,2-diphenyl-1-picrylhydrazyl free radical. Hepatoprotection was evaluated through hepatocyte viability tests using *tert*-butyl hydroperoxide as a stress inducer, (3-(4,5-dimethylthiazol-2-yl)-2,5-diphenyltetrazolium bromide, neutral red uptake, real-time cellular impedance) and mitochondrial function tests. We showed that a 5-min incubation time is sufficient for an optimal bioaccessibility of tea compounds with the highest antioxidative ability, which decreases for longer durations. A 4-h pretreatment of cells with ATE significantly prevented cell death by regulating reactive oxygen species production and maintaining mitochondrial integrity. Pure EGCG, at doses similar in ATE (5–12 µM), was inefficient, suggesting a plausible synergy of several water-soluble tea compounds to explain the ATE beneficial effects.

## 1. Introduction

Tea is the second most consumed beverage worldwide, after water, and is obtained from brewing leaves of *Camellia sinensis*. Tea is classified into five categories according to the degree of fermentation: unfermented (white and green teas), partially fermented (oolong teas), fully fermented (black and pu-erh teas) [1]. Tea is a good source of powerful antioxidant molecules by containing phenolic compounds such as catechins—mainly epicatechin (EC), epigallocatechin (EGC), epicatechin gallate (ECG) and epigallocatechin gallate (EGCG) [2]—and also phenolic acids such as gallic acid (GA) [3] and alkaloids such as caffeine (CAF) [3]. Among these compounds, EGCG, the predominant catechin, has been extensively studied for its antioxidant ability [4]. The type and the amount of antioxidant molecules vary depending on the tea category [5]. Furthermore the bioaccessibility, *i.e.*, the release of such molecules from the leaf matrix into the water during infusion is also variable, depending on brewing temperature (from 50 to 100 °C) and duration (from 2 to 120 min) [6]. *Camellia sinensis* is considered beneficial against various diseases associated with oxidative stress such as liver diseases and more especially non-alcoholic fatty liver disease (NAFLD) [7]. Oxidative stress represents an imbalance between reactive oxygen species (ROS) production and the cellular antioxidant defense system. Excessive production of ROS leads to oxidative stress that can damage all components of the cell including proteins, lipids and DNA [8]. Restoration of the oxidative balance using tea could therefore represent a way to prevent or cure these diseases. We thus aimed to test the activity of antioxidant molecules provided by a mixture of different teas’ leaves (green, oolong and pu-erh) after different brewing durations, compared to pure EGCG, in the prevention of oxidative stress in a primary culture of rat hepatocytes.

## 2. Results

### 2.1. Impact of Brewing Duration on Tea Phenolic Compounds and Caffeine Composition 

**Figure 1:**
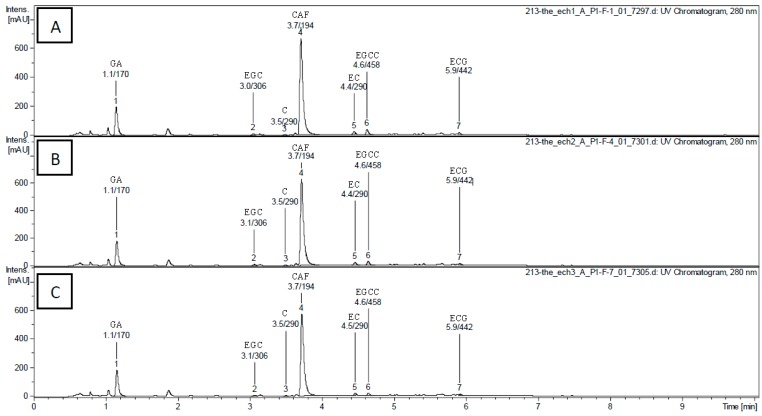
HPLC-DAD/ESI-MS chromatogram of tea extracts ATE-5 (**A**), ATE-15 (**B**) and ATE-30 (**C**) at 280 nm (retention time/molecular weight). The numbered peaks are denoted in Table 2.

The level of total polyphenols of aqueous tea extracts (ATE) decreased significantly by about 10% as the infusion time increased from 5 to 15 (in ATE-5 and ATE-15) to 30 min (in ATE-30) (Table 1), but with no change in the dry extract yield. Several phenolic compounds and caffeine were detected in each ATE (Figure 1), and their concentrations were calculated from the peak area and the calibration curve of each component (Table 2). The main compounds present were caffeine (about 6%–7%) and gallic acid (around 1.3%–1.4%). EGC, EC, EGCG and ECG were also detected in each ATE at concentrations ranging between 0.1% and 1.1%. We observed that gallic acid and caffeine were significantly decreased for ATE-15 (−6.3% and −5%, respectively) and ATE-30 (−7.7% and −10.8%, respectively) compared to ATE-5. We found that EGC, EC, EGCG and ECG were significantly decreased after 30 min of infusion compared to 5 min (−35%, −23.8%, −63.6%, −54.5%, respectively). Fifteen min of infusion induced only a trend of decrease of EC. Global proportion of antioxidants was in accordance with the mixture of teas used, catechins being mainly provided by green and oolong teas, gallic acid by pu-erh tea, and caffeine being equally provided by each tea [3].

**Table 1:** Yield percent and total polyphenols of aqueous tea extracts.

Sample ^1^	Mean of Dry Extract ^2^ (g)	Yield (%)	Total Polyphenols ^2–3^ (g/100g Dry Extract)
ATE-5	4.00 ± 0.15	20.02	20.9 ± 0.3 ^a^
ATE-15	3.98 ± 0.10	19.88	20.2 ± 0.1 ^a^
ATE-30	3.85 ± 0.26	19.23	18.9 ± 0.3 ^b^

^1^ Aqueous Tea Extracts (ATE-5, ATE-15 and ATE-30) were obtained by infusing 20 g of tea leaves in 200 mL boiling water for 5, 15 and 30 min, respectively. The infusions were then lyophilized and the freeze-dried extracts were weighed and quantified for their total polyphenols content (gallic acid equivalents); ^2^ Results are means ± SEM of three independent experiments; ^3^ Means with different superscript letters are significantly different (ANOVA with Bonferroni’s multiple comparison test, *p* < 0.05).

**Table 2:** ESI-MS/MS fragments of the compounds identified in the aqueous tea extracts.

Retention Time (min)	Molecular Mass (Da)	[M − H]^−^/[M + H]^+^	Fragment Ions in Negative Mode	Compound Structures ^1^	Compound Content (g/100 g of Dry Extract) ^1–3^		
					ATE-5	ATE-15	ATE-30
1.1	170	169/-	125	GA	1.43 ± 0.01 ^a^	1.34 ± 0.03 ^b^	1.32 ± 0.02 ^b^
3.0	306	305/-	179, 125	EGC	0.20 ± 0.01 ^a^	0.19 ± 0.02 ^a^	0.13 ± 0.01 ^b^
3.5	290	-/291	-	C	<LQ	<LQ	<LQ
3.7	194	-/195	138	CAF	6.87 ± 0.02 ^a^	6.56 ± 0.05 ^b^	5.87 ± 0.03 ^c^
4.4	290	-/291	-	EC	0.42 ± 0.04 ^a^	0.35 ± 0.02 ^a,b^	0.32 ± 0.04 ^b,c^
4.5	458	457/-	305, 169	EGCG	1.10 ± 0.20 ^a^	0.88 ± 0.03 ^a^	0.40 ± 0.10 ^b^
5.9	442	441/-	289	ECG	0.33 ± 0.03 ^a^	0.29 ± 0.02 ^a^	0.15 ± 0.04 ^b^
				Total catechins	2.05 ± 0.28 ^a^	1.71 ± 0.09 ^a^	1.0 ± 0.2 ^b^
				Total	10.3 ± 0.3 ^a^	9.6 ± 0.1 ^b^	8.2 ± 0.2 ^c^

^1^ ATE, Aqueous Tea Extract; C, Catechin; CAF, Caffeine; EC, Epicatechin; ECG, Epicatechin-3-*O*-gallate; EGC, Epigallocatechin; EGCG, Epigallocatechin-3-*O*-gallate; GA, Gallic acid; LQ, limit of quantification; ^2^ Results are expressed as means ± SEM of three parallel measurements; ^3^ For a given row, means with different superscript letters are significantly different (ANOVA with Bonferroni’s multiple comparison test, *p* < 0.05).

### 2.2. Antioxidant Activity of ATE Compounds

**Figure 2:**
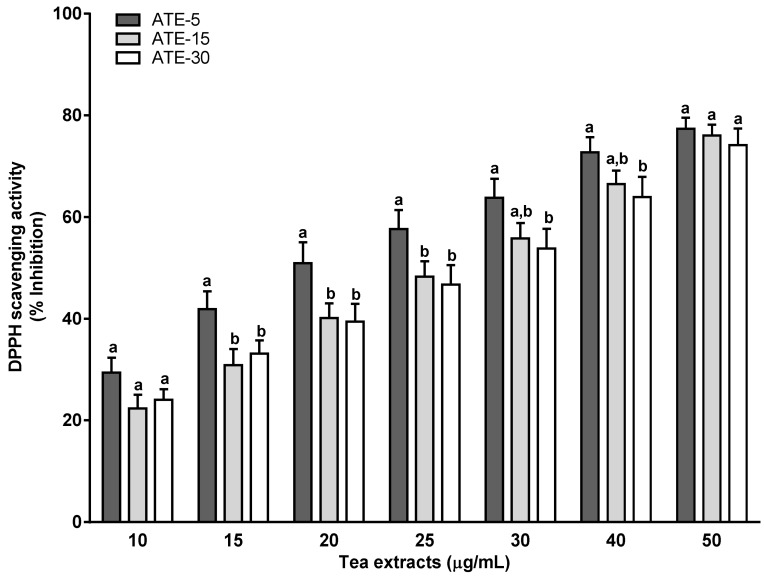
Antioxidant activity of tea extracts ATE-5, ATE-15 and ATE-30 using DPPH scavenging activity. The DPPH radical-scavenging activity was calculated as percent inhibition according to the following equation: % Inhibition = [(Absorbance_control_ − Absorbance_sample_)]/(Absorbance_control_)] × 100. Results are means ± SEM (*n* = 3). For a given concentration, means with different superscript letters (a, b) are significantly different (*p* < 0.05).

The scavenging activity (% inhibition) against 2,2-diphenyl-1-picrylhydrazyl free radical (DPPH) of ATE-5 was significantly higher (about 5%–10%) than those of ATE-15 from 10–25 µg/mL and of ATE-30 from 10–40 µg/mL (Figure 2). Thus, ATE-5 demonstrated a stronger DPPH radical scavenging activity with an IC_50_ value of 20 µg/mL, whereas the IC_50_ values were higher for ATE-15 and ATE-30 (between 25–30 µg/mL). For the concentration 50 µg/mL (corresponding to 9.45–10.45 µg of total polyphenols per mL) values reached a plateau and were similar for the three ATE (76%–80% inhibition).

### 2.3. Effect of ATE or EGCG on Viability of Rat Hepatocytes

**Figure 3:**
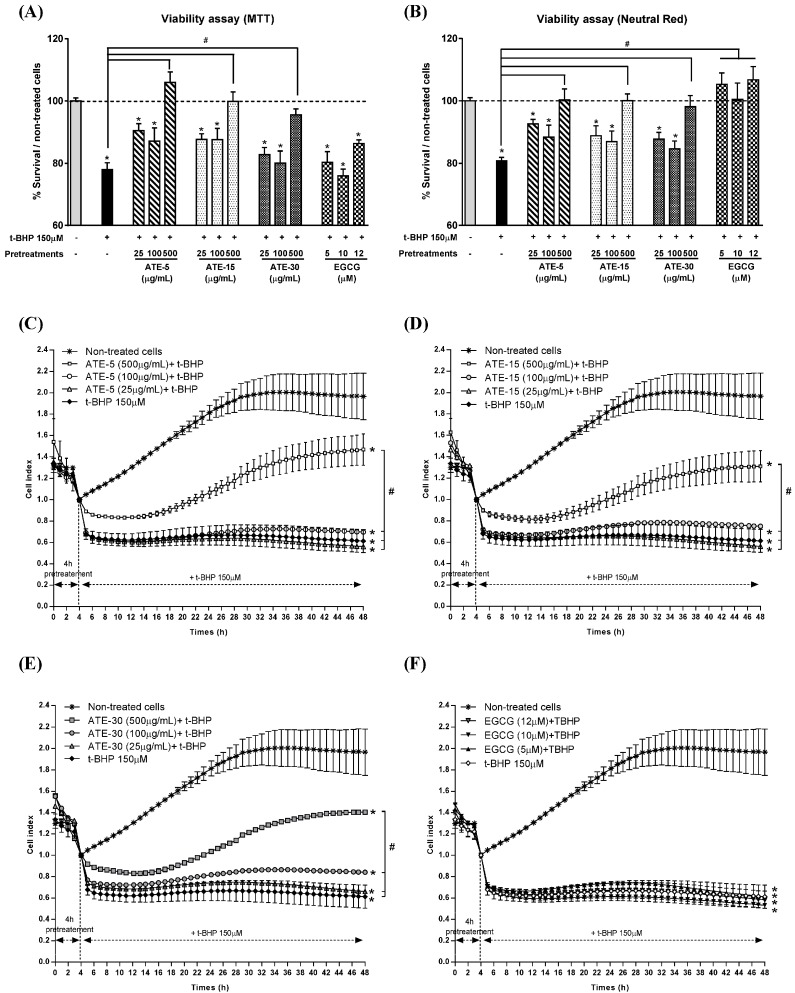
Effect of ATE or EGCG on the viability of rat hepatocytes. Cell viability was assessed by the MTT (**A**) and neutral red uptake (NRU) test (**B**) after 4 h pretreatment with 25, 100 and 500 µg/mL tea extracts or 5, 10 and 12 µM EGCG followed by 1 h exposure to 150 µM *t*-BHP. MTT and NRU results are presented as % viability over non-treated cells (mean ± SEM of three separate experiments; note: * *p* < 0.05 when compared to non-treated cells, ^#^ *p* < 0.05 when compared to non-pretreated + *t*-BHP treated cells; − indicates the absence of *t*-BHP or ATE, and + indicates the presence of *t*-BHP, in the culture medium). For cell impedance tests (**C**–**F**), cells were pretreated for 4 h with 25, 100 and 500 µg/mL of tea extracts or 5, 10 and 12 µM EGCG, and incubated for 44 h with *t*-BHP at 150 µM. Cell impedance was measured in real-time (RTCI) and cell index was normalized prior the addition of *t*-BHP. Results are means ± SEM for triplicates of one experiment and are representative of three independent experiments (note: * *p* < 0.05 when compared to non-treated cells, ^#^ *p* < 0.05 when compared to non-pretreated + *t*-BHP treated cells).

A 1 h treatment with 150 µM *tert*-butyl hydroperoxide (*t*-BHP) caused a significant 22% loss in cell viability when using the 3-(4,5-dimethylthiazol-2-yl)-2,5-diphenyltetrazolium bromide (MTT) assay (Figure 3A) and of 19% by using the Neutral Red Uptake (NRU) assay (Figure 3B), in comparison with the non-treated cells. Pretreatments for 4 h with 500 µg/mL of ATE prevented the decrease of cell viability, whatever the brewing duration. The other tea extract concentrations (25 and 100 µg/mL) were ineffective. The MTT test showed no protective effect of EGCG (at 5, 10 and 12 µM), whatever the concentration used (Figure 3A), while the NRU test indicated that EGCG pretreatment fully prevented the decrease of the cell viability induced by *t*-BHP identically for the three concentrations tested (Figure 3B).The real-time cellular impedance (RTCI) analysis performed continuously over 48 h (Figure 3C–F) revealed a quick significant decrease of the cell index after *t*-BHP treatment (150 µM) that remained low over 48 h. This decrease was similarly partially prevented only by 500 µg/mL of tea extract from ATE-5 (Figure 3C), ATE-15 (Figure 3D) and ATE-30 (Figure 3E). Furthermore, the cell index increased over-time probably due to the reconstitution of cell layer. Pure EGCG failed to prevent the cell index decrease (Figure 3F). Of note, we observed no toxic or proliferative effect of ATE or EGCG, when administered alone at the concentrations used, on rat hepatocytes (pretreated cells without *t*-BHP) via microscopic observations, NRU and MTT assays, and RTCI (data not shown).

### 2.4. Effect of ATE or EGCG on Mitochondrial Superoxide Anion (O_2_^−^) Production and on Mitochondrial Functionality

**Figure 4:**
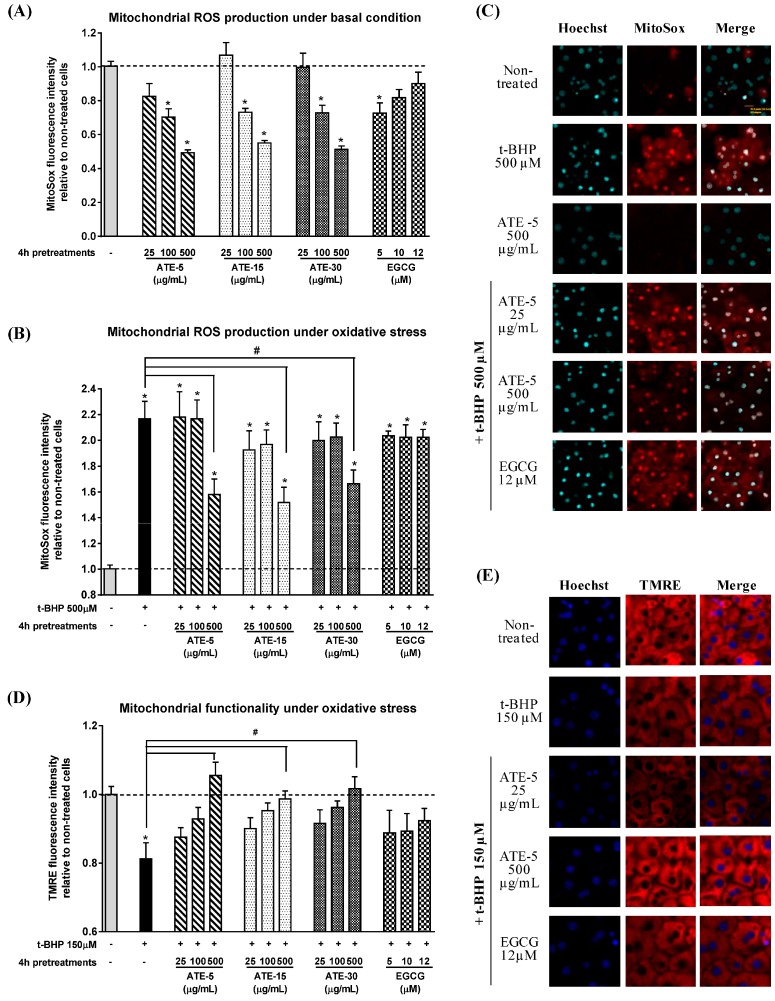
Effect of ATE or EGCG on mitochondrial superoxide anion (O_2_^−^) production and on mitochondrial functionality. Cells were seeded onto 96-well E-plates, pretreated for 4 h with various concentrations of tea extracts (25, 100 and 500 µg/mL) or EGCG (5, 10 and 12 µM), then treated for 1 h with *t*-BHP at 150 µM. Cells were labeled with Hoechst 33342 and MitoSox (**A**–**C**) or TMRE (**D**,**E**) for 30 min before being read on the ArrayScanXTI. Mitochondrial superoxide (O_2_^−^) content (**A**,**B**) was calculated from the MitoSox fluorescence intensity following the “compartmental analysis protocol”. Mitochondrial functionality (**D**) was calculated from the TMRE fluorescence intensity following the “compartmental analysis protocol”. Error bars indicate the mean ± SEM of triplicate determinations in three independent experiments (note: * *p* < 0.05 when compared to non-treated cells, ^#^ *p* < 0.05 when compared to non-pretreated + *t*-BHP treated cells). Objective magnification ×20. General note: − indicates the absence of *t*-BHP or ATE, and + indicates the presence of *t*-BHP, in the culture medium.

The mitochondrial O_2_^−^ production, as a marker of the main ROS generated by the rat hepatocytes [9], was evaluated by MitoSox staining (Figure 4A–C). ATE pretreatments decreased O_2_^−^ production in unstressed hepatocytes (Figure 4A,C). Indeed, by comparison to non-treated cells, we observed a significant reduction of O_2_^−^ production under basal condition, calculated from the decrease of the fluorescence of the MitoSox red staining, with ATE-5, ATE-15 and ATE-30 at 100 μg/mL (−29.7%, −26.9% and −27.2%, respectively) and at 500 μg/mL (−50.7%, −45.0% and −48.7%, respectively). The 25 µg/mL dose was ineffective. A significant 27.4% decrease in O_2_^−^ production was also observed with 5 µM EGCG pretreatment, while only a trend was obtained with higher concentrations (10 and 12 µM). Then, we investigated whether ATE pretreatments prevent the *t*-BHP-induced oxidative stress of mitochondria. *t*-BHP induced a 2.2-fold significant increase in mitochondrial ROS production compared to non-treated cells (Figure 4B,C). This induction was in part prevented by 500 µg/mL of all ATE pretreatments, leading to a final oxidant stress increase of about 1.6-fold compared to the non-treated condition, *i.e.*, a protection of about 50%. The lowest doses did not prevent *t*-BHP-induced mitochondrial oxidative stress, meaning the level of antioxidant compounds was not sufficient to protect cells against such a high *t*-BHP concentration (high stress condition). Pure EGCG failed to protect mitochondria from *t*-BHP-induced oxidative stress at any concentration. Since accumulation of O_2_^−^ within the mitochondria following increasing oxidative stress leads to compromised mitochondrial functionality by first reducing membrane integrity [10], we further investigated whether ATE or EGCG pretreatments protect the mitochondrial functionality evaluated by tetramethylrhodamine ethyl ester (TMRE) staining. As demonstrated by the 20% decrease of the TMRE red staining (Figure 4D,E) a 1-h treatment with 150 µM t-BHP caused a significant loss of mitochondrial functionality compared to the non-treated cells. Pretreatment with ATE, but only at the dose of 500 µg/mL and independently of the infusion time, resulted in normal mitochondrial functionality despite the *t*-BHP-induced oxidative stress, whereas basal mitochondrial functionality (absence of *t*-BHP) was not affected by ATE treatments (data not shown). Pure EGCG did not prevent the decrease in mitochondrial functionality induced by *t*-BHP.

## 3. Discussion

*Camellia sinensis* is considered beneficial against various diseases associated with oxidative stress such as liver diseases and more especially NAFLD [7]. Thus the aim of the present study was firstly to evaluate the effect of brewing duration of a mixture of teas’ leaves (green, oolong and pu-erh) on the bioaccessiblity and the antioxidative properties of the main phenolic and alkaloïd compounds, and secondly, to test their hepatoprotective effects. We have chosen a blend of three teas representative of the conventional processes used in the preparation of tea leaves (unfermented, partially fermented and fully fermented) to obtain a natural source of a larger variety of antioxidants as possible.

We demonstrate that a 5 min infusion time is sufficient to reach maximal bioaccessibility of caffeine and phenolic compounds in tea, and a further increase of the brewing duration (15 or 30 min) results in no change, or even in a decrease, of their concentrations. ATE-5 consistently exhibited stronger free radical scavenging capacity as evaluated with the DPPH biochemical assay. This latter result is informative even if obtained by using only DPPH assay. Indeed, DPPH is a widely used method to assess antioxidant activity of food compounds by measuring free radical scavenging capacity *in vitro,* but diverse other methods of interest are available and their relevance and exact meanings are still the subject of much debate, as reviewed [11,12]. Nevertheless, the DPPH method has been described to be strongly correlated with other antioxidant assays such as ABTS (2,2′-azinobis(3-ethylbenzothiazoline-6-sulfonate)) (R = 0.91), FRAP (ferric reducing antioxidant potential) (R = 0.82) and ORAC (oxygen radical absorption capacity) (R = 0.85) assays, and is commonly used for evaluating the antioxidant potential of plant and tea extracts *in vitro* [13]. Thus our observations suggest that antioxidant capacity could be linked to infusion time and consequently specifically to polyphenols and caffeine bioaccessibility. The duration of brewing (from 2 to 120 min) is an important factor affecting the yield of phenolic compounds and caffeine extracted in water [14,15], and durations as long as 10 or 30 min led to higher concentration of phenolic compounds [6], which was not the case in our study while the yield of matter was not affected. This could be explained by our water-to-tea leaves ratio (10 mL:1 g) being limitative for further extraction over time as an optimal ratio for catechins extraction could be 50 mL:1 g [6]. In addition, catechins decreased after 30 min of infusion, possibly due to their epimerization, oxidation and degradation under high extraction temperatures [15]. Moreover, it is generally recognized that polyphenols, and mainly catechins, are responsible for the antioxidant activity of tea. Indeed, these molecules are efficient free-radical scavengers partly due to their one-electron reduction potential, *i.e.*, the ability to act as hydrogen or electron donors [16]. Our study is thus in agreement with the literature [17] demonstrating a relationship between the antioxidant activity and the number of hydroxyl groups as our ATE-5 is the tea extract containing the highest EGCG (eight hydroxyl groups) and EGC (seven hydroxyl groups) contents.

Because previous studies have shown that tea polyphenols can modulate pro-oxidant and antioxidant cellular enzymes, the chemical-based antioxidant potencies tested above could not guarantee cellular biological activity [18]. Therefore, it was necessary to complete this first observation by cellular *in vitro* assays. Thus, we tested the effects of the three ATE or pure EGCG, the major catechin present in our ATE, on oxidative stress either under basal condition (un-stressed hepatocytes) or exogenously induced by *t*-BHP in isolated rat hepatocytes in primary culture. The exposure to *t*-BHP is well known to simulate augmented oxidative stress causing cell death by stimulating ROS overproduction within the cells, leading to an increase of lipid peroxides and depleting the cell in glutathione peroxidase (one of the key cellular self-defense systems for scavenging ROS) leaving the reactive intermediates formed free to attack membrane phospholipids, proteins and nucleic acids [19]. Consequently, a high alteration of the mitochondrial membrane integrity, ascertainable by a reduction of the mitochondrial membrane potential, occurs with impairment of the respiratory chain leading to the generation of more ROS through a vicious cycle [19]. We thus axed our experiments on cell viability, on ROS generation by the rat hepatocyte and especially on the mitochondrial superoxide anion (O_2_^−^) production as a strong stress oxidant inducer, and on mitochondrial membrane integrity, linked to membrane potential, for exploring the hepato-protective effects of our tea.

As regards to cell viability, all three tea extracts at 500 µg/mL (providing 5.15 to 4.1 µg total catechins, caffeine, and gallic acid per cell well) demonstrated a significant protective effect against *t*-BHP elicited cell death, independently of the infusion time. Three different types of test confirmed this effect. Our observation is concordant with previous *in vitro* studies showing a beneficial impact of tea compounds on hepatocyte viability [20]. Since infusion time was shown to decrease catechin content in ATE in parallel to a decrease in global antioxidant activity, the same efficiency observed with the three highest ATE doses on the prevention of hepatocytes death is probably related to other bioactive molecules in tea. Surprisingly, pure EGCG, even at the highest and non-toxic dose selected (12 µM), failed to prevent hepatocytes death as indicated by the MTT test, but seemed effective when using the NRU test. To explain this interesting difference, we hypothesized that EGCG could disrupt the lysosomal activity as already observed in different cell lines [21], making the NRU assay unsuitable (false positive). Indeed, this test is depending on the ability of neutral red to incorporate into the lysosomes of living cells. Moreover, this hypothesis is supported by the RTCI data showing, as the MTT test did, that EGCG was unable to prevent cell death induced by *t*-BHP. 

When focusing on mitochondria, firstly our tea mixture displayed interesting properties regulating the basal oxidative stress induced by normal hepatocyte mitochondrial activity. Indeed, it was estimated that 0.4% to 4.0% of all oxygen consumed in mitochondria during normal oxidative phosphorylation is transformed into superoxide anion (O_2_^−^) [22], and our medium and highest doses of tea were efficient enough to maintain down by 30% to 50% the concentration of this strong stress oxidant inducer. Secondly, in a high oxidative stress condition the highest concentration of tea extracts, *i.e.*, a sufficient amount of antioxidant compounds (about 100 µg of total polyphenols per mL plus 30 µg of caffeine), has however been necessary to prevent partially the overproduction of ROS within the mitochondria. Even with a decrease of only 50% in O_2_^−^, this limitation was sufficient enough to maintain a mitochondrial membrane full integrity *i.e.*, ensuring a normal membrane potential, with at final a better viability of hepatocytes. Our findings are in accordance with the fact that ROS overproduction is the starting point for mitochondrial dysfunction [22] consecutively implicated non only in the development of liver diseases such NAFLD [9,23] but also in a wider range of diseases such as cardiovascular, neurodegenerative, and skin aging [24,25,26,27], designating the mitochondria as a central target to prevent or treat these diseases. Our study contributes to reinforce previous works reporting that tea compounds are good candidates to protect mitochondria functionality for such purposes by regulating the mitochondrial membrane integrity *i.e.*, blocking the decline of mitochondrial membrane potential [22,28,29] and by improving the respiratory performance measured with the oxygen consumption rate [30]. The leakage of the mitochondrial membrane (so a decrease in membrane potential by loss of integrity), consequent to accumulation of ROS, has been reported to trigger the mitochondria dependent apoptotic signal pathways (such as decrease in Bcl-2 and increase in Bax and Bad; release of cytochrome c into the cytosol in association with up-regulation of caspase-9, Apaf-1 and caspase-3) programming the cell death [28,31,32]. Regarding plausible mechanisms of action, we can postulate that our tea extracts maintained hepatocytes alive by preventing this cascade of events as reported for another type of herbal tea [28]. Thirdly, we did not detect differences between 500 µg/mL ATE-5, -15 and -30 with regard to oxidative stress levels despite a decrease in polyphenols content due to infusion-time. This observation could be explained by the presence of other antioxidant compounds in tea, not determined in this study, which could be more stable and thus less degraded by infusion time, and at final much more specifically responsible for sequestration of superoxide anions. Indeed, tea is also a rich source of theaflavins and thearubigins, products of biotransformation of catechins [33]. Theaflavins, as well as catechins, have demonstrated powerful antioxidant activities [34] and was previously found to be more effective than catechins in abrogating O_2_^−^ production in macrophage cell culture [35], in protecting cardiomyocytes from oxidative damage [36] and in suppressing ROS production through powerfull inhibition of the xanthine oxydase in HL-60 cells [37]. 

Finally, EGCG did not prevent hepatocytes against either cell death or oxidative stress induced by *t*-BHP. These results are in contradiction with previous data demonstrating that cellular EGCG was able to counteract oxidative damage induced by *t*-BHP in a rat hepatoma cell line [38]. However, EGCG was used at higher concentration (50 µM, for 1 h incubation time) compared to the concentrations used in our study (5 to 12 µM). EGCG could therefore have a beneficial action against oxidative damage induced by *t*-BHP but for a concentration that is not representative of the level of molecule present in our tea mixture extract. Furthermore, it was shown that EGCG used at low dose (<10 µM, 24-h incubation time) reduced the production of ROS in primary rat hepatocytes under basal conditions (as found herein), while higher doses (≥10 µM) displayed high toxicity (increase of ROS generation, decline in mitochondrial membrane potential, apoptosis) [30]. In addition, green tea extract was reported to induce *in vitro* hepatotoxicity when used at a concentration of 1–3 mg/mL while concentrations from 100–500 µg/mL enhanced cell viability [39]. Such hepatotoxicity induced by high doses of catechins, especially EGCG, has raised a safety concern for green tea consumption at high quantity (infusion or concentrated extract supplements) in humans especially in clinical conditions predisposing to liver injury (pre-existing hepatic steatosis, use of potentially hepatotoxic drugs such as statins, genetic polymorphisms) [40]. Finally, our experimental ATE doses (25, 100 and 500 µg/mL) were chosen to mimic plasma level of polyphenols in rodent experimental conditions that was reported to be up to 10 µM after oral intake of pure EGCG [41], while bioavailability studies in humans demonstrated that maximum level of EGCG reached in plasma was about 1 μM after green tea consumption (corresponding to about two cups of tea/day) [42]. Thus in our study, rat hepatocytes were exposed to 5.5 to 7.2 µM of EC, 4.3 to 12 µM of EGCG and 1.7 to 3.7 µM of ECG, respecting the physiological conditions in the experimental rodent model, and being in the range of concentrations considered as “safe”. This strengthens the validity of our findings *in vitro* by suggesting plausible beneficial effects *in vivo*. 

In conclusion, our study shows that a five minute infusion time was sufficient to achieve maximal bioaccessibility and benefits of tea compounds from leaves of green/oolong/pu-erh teas, but that chemical-based antioxidant potencies (using the classical DPPH assay) cannot guarantee further cellular biological activity. Finally, only the highest but still “physiological” concentrations of ATE, whatever the brewing duration, displayed hepatoprotective effects against *t*-BHP-induced oxidative stress by sufficiently regulating the mitochondrial ROS production to a certain level to maintain mitochondrial global function and integrity, leading to the prevention of hepatocyte death. These effects were not directly linked to the EGCG fraction present, that failed *per se* to counteract *t*-BHP toxicity, but rather to a plausible synergistic effect between several water soluble tea compounds yet to be further characterized. The authors are aware of the limitations of this *in vitro* study *i.e.*, the lack of measurements that would have characterized more in details the oxidative stress outcomes such as lipid/protein peroxidation, DNA’s damage, expression of key genes involved in protection against oxidative stress, and the measurement of the respiratory activity assessing more deeply the mitochondrial function, however, the present study represents a promising starting point for a future investigation in a diet-induced NAFLD rat model in order to confirm the beneficial impact of this blend of teas in the protection of the liver, and to explore more deeply directly *in vivo* the underlying mechanisms of action.

## 4. Experimental Section

### 4.1. Chemicals

Neutral red, 3-(4,5-dimethylthiazol-2-yl)-2,5-diphenyltetrazolium bromide (MTT), epigallo-catechin-3-*O*-gallate (EGCG), 2,2-diphenyl-1-picrylhydrazyl free radical (DPPH), *tert*-butyl hydroperoxide (*t*-BHP) and dimethyl sulfoxide (DMSO) were purchased from Sigma-Aldrich (Saint Quentin Fallavier, France). Type CLS2 collagenase was from Roche Applied (Meylan, France). William’s E medium, penicillin/streptomycin, trypsin/EDTA, fetal bovine serum and mitochondrial superoxide indicator (MitoSox) were purchased from Life Technologies (Saint Aubin, France). Tetramethylrhodamine ethyl ester (TMRE) was from Interchim (Montluçon, France). Ethanol and acetic acid were from Prolabo (Fontenay sous Bois, France). The human insulin was from Novordisk (Bagsvaerd, Denmark).

### 4.2. Tea Origin and Aqueous Extract Preparation

Hao Ling^®^, is a blend of green, oolong and pu-erh tea leaves coming from Zhejiang, Fujian and Yunnan provinces (China), respectively. This blend of teas, commonly consumed in France and in other countries (Canada, Switzerland, Belgium, Sweden, Italy, UK, Luxembourg), is elaborated following a confidential manufacturing process by the French company “Thés de la Pagode” (Paris, France). The tea leaves had been harvested in spring and summer. Fresh leaves were then processed as follows using a routine and very well-controlled industrial procedure: withering and drying for green tea, rolling, short oxidation and drying for oolong tea, and rolling, sun drying, piling and drying for pu-erh tea. The leaves of these teas were ground to obtain a homogeneous fine powder (particles < 1 mm). The infusions were prepared by pouring 200 mL of distilled water at 100 °C on 20 g of tea leaves mixture powder and brewing for 5, 15 and 30 min. The infusions were filtered and lyophilized using Heto PowerDry LL1500 (Thermo Electron, Beverly, MA, USA), and the freeze-dried aqueous tea extracts (designated as ATE-5, ATE-15, ATE-30) were stored at −20 °C until analysis. Each dried ATE was diluted appropriately with water or cell culture medium according to each specific assay.

### 4.3. Characterization and Quantification of the Tea Extracts Compounds

Total phenolic content of ATE (0.020–0.030 g extracts/2 mL distilled water) was determined by the Folin-Ciocalteu method using a standard solution of gallic acid as calibration curve [43]. Results are expressed as gallic acid equivalents in g/100 g dry matter. Further characterization and quantification of ATE compounds was performed by HPLC-DAD/ESI-MS (Esquire 3000, Bruker Daltonics, Bremen, Germany). The compounds separation was carried out with a C18 column (2.1 mm × 100 mm i.d., 1.8 µm film thickness, Agilent, Santa Clara, CA, USA) kept at 25 °C. For analysis, 1 µL of each ATE solution (30 mg/6 mL distilled water) filtered through nylon filter 0.45 μm was injected. The solvents used were water/0.1% methanol (mobile phase A) and acetonitrile/0.1% methanol (mobile phase B). The gradient elution program using a flow rate of 0.4 mL/min was: 7% B from 0 to 3 min; 14% B up to 8 min; 35% B up to 8.8 min; 50% B up to 9 min; 100% B up to 10.2 min and 7% B until 13.2 min, phase A being used to reach 100%. At the output of the diode array detector, the effluent was injected into the mass spectrometer. Analyses were performed in positive mode and/or negative mode. Capillary voltage was −4.6 kV, the nebulizer was driven by compressed air (regulated to 40 psi) at 10 L dry gas/min and the capillary was set at 365 °C. The LC-MS spectra were acquired in “Full Scan” mode on the whole mass range (*m*/*z*) of 100 to 1400. The data were processed by the Hystar version 3.0 software (Bruker Daltonics, Bremen, Germany). The identification and quantification of compounds were performed with standard solutions of catechins (EGC, EC, EGCG and ECG), gallic acid, and caffeine (in acetonitrile/water, 1:1, *v*/*v*), and using calibration curves for each standard molecule. 

### 4.4. Antioxidant Capacity of ATE Compounds

The antioxidant activity of ATE was determined by measuring their scavenging ability against DPPH free radical as described [44]. Reconstituted ATE (50 µL of a 1 mg/mL distilled water solution) mixed with 0.1 mM methanolic DPPH solution (100 µL) were incubated for 1 h at room temperature into darkness, and the optical absorbance at 525 nm was measured using ELx808 Absorbance Reader (Biotek, Colmar, France). A control with no extract was run in parallel. The DPPH radical-scavenging activity was calculated as percent inhibition according to the following equation: % Inhibition = [(Absorbance_control_ − Absorbance_sample_)]/(Absorbance_control_)] × 100. The concentration of the ATE required for inducing a 50% decrease in initial DPPH concentration (IC_50_) was calculated. Measurements were performed in triplicate.

### 4.5. Isolation, Cultivation of Rat Hepatocytes and Treatments

The national guidelines for care and use of research animals were followed (agreement number A 13823, French Ministry of Agriculture). Hepatocytes were freshly isolated from *Rattus* *norvegicus* OFA male rats weighing 180 to 200 g (Iffa Credo, L’isles d’Arbesle, France) by collagenase perfusion of the liver [45]. The hepatocytes were suspended in William’s E medium supplemented with 10% foetal bovine serum, 50 UI/mL penicillin, 50 μg/mL streptomycin and 0.1 UI/mL insulin, and plated in collagen-coated 96-well plates (E-plates RTCA, San Diego, CA, USA, 2.5 or 3 × 10^4^ cells/well depending on the experiment). The cells were cultured 24 h at 37 °C in a humidified atmosphere of 95% O_2_ and 5% CO_2_. The medium was replaced by a serum-free medium supplemented with hydrocortisone hemisuccinate (1 µM) and bovine serum albumin (240 μg/mL). Then, rat hepatocytes in primary culture were pretreated for 4 h with 100 µL of various concentrations of ATE (0 to 0.5 mg/mL) or EGCG (5, 10 and 12 µM chosen to mimic the amount present in ATE-5, ATE-15 and ATE-30, respectively). After the 4-h, the cell medium was removed and replaced by a medium free of ATE or EGCG, and the hepatocytes were treated with the exogenous oxidative stress inducer *t*-BHP (DMSO solution) using 150 µM or 500 µM for 1 h or 44 h, depending on the experiment. Such doses were selected after testing several concentrations (from 150 µM to 1 mM) in order to generate sufficient oxidative stress without killing all cells (data not shown). Then, cell viability tests and mitochondrial assays were performed. Control *i.e.*, “non-treated” cells consisted of medium, water or DMSO depending on the experiment. DMSO did not interfere with the experiment when present (data not shown). Cells were also incubated only with ATE or EGCG without *t*-BHP to verify any toxic and proliferative effect of the antioxidants.

### 4.6. Hepatocyte Viability Tests

The cell viability was determined by two classical tests, *i.e.*, the MTT and NRU assays, and real-time cellular impedance (RTCI). The conversion of the MTT to formazan was measured as previously described [46]. The NRU procedure is a colorimetric measurement of the ability of viable cells to incorporate and bind the supravital dye neutral red in the lysosomes. A neutral red solution (final 4 mg/mL) was added to each well after the cell medium was removed. After 2 h incubation, the neutral red was removed, the plates were incubated with destain to solubilize the neutral red uptaken by the cell lysosomes, and the optical density was measured at 550 nm. RTCI was measured in 96 well-plate E-plates with 80% of their bottom surface covered with gold microelectrodes using a special analyzer (RTCA, xCELLigence^®^ system, Roche Applied Science, Mannheim, Germany and ACEA Biosciences, San Diego, CA, USA) as previously described [47]. RTCI was measured in each well (cell index values) and the signal was observed through the integrated RTCA software (Biosciences, San Diego, CA, USA, v2.0). Each curve was obtained from three wells by plate and was representative of the curves obtained from three independent experiments. This technique monitored the overall cellular status of adherent cells in culture, *i.e.*, variations in cell number and cytomorphology in a non-invasive environment continuously over 48 h.

### 4.7. Measurement of Mitochondrial Superoxide Anion (O_2_^−^) and of Relative Mitochondrial Activity

Mitochondrial superoxide anion was measured using MitoSox red staining that specifically binds to O_2_^−^ within the cells. The relative mitochondrial activity was measured by TMRE membrane potential staining red probe, a higher staining being indicative of a high activity of the mitochondria. Probes fluorescence was measured with an ArrayScan XTI high Content Analysis Reader (Cellomics Inc., Pittsburgh, PA, USA) using the High Content Screening software, following a procedure already published [47]. Briefly, the stock solutions of probes were diluted with HANK’s medium (5 µM for MitoSox and 25 nM for TMRE) and 100 µL were added to each well and incubated with the nuclear marker Hoechst 33342 (2.5 µg/mL final) at 37 °C for 30 min. The detection was performed using the “compartmental analysis protocol” bio-application after replacement of medium-containing fluorochromes by PBS 0.01 M. An objective of 20× was used for the imaging analysis.

### 4.8. Statistical Analysis

Data are mean values ± standard error of the mean (SEM). Statistical analysis was performed by one-way analysis of variance (ANOVA) with Bonferroni’s multiple comparison test. Results were considered significant for *p* < 0.05. 
